# Stepping Out: A Novel Pilot Falls Prevention Program for Individuals With Mild Cognitive Impairment

**DOI:** 10.1093/geroni/igaa057.1568

**Published:** 2020-12-16

**Authors:** Barbara Fischer, Allison Midden, Aundrea Hoffmann, Lynn DeWitt, Kathryne Kohlman, Lindy Clemson, Katherine Sherman, Jane Mahoney

**Affiliations:** 1 Clement J. Zablocki VA Medical Center, Milwaukee, Wisconsin, United States; 2 Clement J. Zablocki VA Medical Center, Green Bay, Wisconsin, United States; 3 Unversity of Sydney, Sydney, Australia; 4 University of Wisconsin - Madison, Madison, United States

## Abstract

Objectives: Falls are the leading source of accidental injury and hospitalization among adults over the age of 65. Relative to people with intact cognition, individuals with cognitive impairment are at increased risk for falls; however, few falls prevention programs exist to specifically reduce and prevent falls in this population. To address this issue, we developed a novel, multifactorial, cognitively-based falls prevention program, Stepping Out. Based on the popular and effective evidenced-based program, Stepping On, Stepping Out was modified and tailored to the learning needs of individuals with Mild Cognitive Impairment (MCI). We hypothesized that older adults with MCI would find the program understandable, and that program participants would demonstrate reduced falls. Methods: Sixteen veterans, mean age of 77.5, diagnosed with MCI and at increased risk for falls participated in Stepping Out. Falls were collected for the six months prior to intervention and the six months during and after program participation. All participants completed post-program evaluations. Falls incidence was compared using a Wilcoxon paired signed rank test. Results: Stepping Out was found to be feasible and comprehensible by all participants. Program participants exhibited significantly reduced falls, with median reduction of two falls (p = 0.0020), and a range of zero to 12 falls. Discussion: With appropriate modifications, individuals with MCI were able to benefit from a cognitively-based falls prevention program and to reduce accidental falls incidence. Falls are an important and feasible target to address among individuals with cognitive impairment.

